# Competition between elasticity and adhesion in caterpillar locomotion

**DOI:** 10.1098/rsif.2024.0703

**Published:** 2025-04-23

**Authors:** Mario Argenziano, Massimiliano Zingales, Arsenio Cutolo, Emanuela Bologna, Massimiliano Fraldi

**Affiliations:** ^1^ Department of Engineering, University of Palermo, Palermo, Sicily, Italy; ^2^ Department of Structures for Engineering and Architecture, Universityof Naples "Federico II", Naples, Campania, Italy; ^3^ Département de Physique, LPENS, École Normale Supérieure-PSL, Paris, France

**Keywords:** crawling gait mechanics, interplay elasticity–adhesion, caterpillar locomotion

## Abstract

In recent years, there has been a growing interest in understanding animals’ locomotion mechanisms for developing bio-inspired micro- or nano-robots capable of overcoming obstacles and navigating in confined environments. Among non-pedal crawlers, caterpillars exhibit one of the most stable and efficient gait strategies, utilizing muscle contractions and substrate grip. Although several approaches have been proposed to model their locomotion, little is known about the competition between body elasticity and adhesion, which we demonstrate playing a central role in crawling gait. Preliminarily, experimental observations and measurements were performed on *Pieris brassicae* larvae, gaining insights into fundamental features characterizing caterpillar locomotion and estimating key geometrical and mechanical parameters. A minimal but effective one-dimensional discrete model was thus conceived to capture all the relevant aspects of the movement. Inter-mass springs model the deformable body units, Winkler-like constraints with an adhesion threshold reproduce elastic interactions and attaching/detaching events at prolegs-substrate interface, and a triggering muscle contraction initiates the larva’s crawling cycle, generating the observed travelling wave. After demonstrating theoretically that caterpillars move obeying quasi-static laws, we proved robustness of the proposed approach by showing very good agreement between theoretical outcomes and experimental evidence, so paving the way for new optimization strategies in soft robotics.

## 1. Introduction

A wide spectrum of animal species and biological organisms exploits the crawling gait as one of the possible locomotion mechanisms, from earthworms, snails and Lepidoptera at larval stage to migrating cells and amoebae that change shape by extending and retracting pseudopods [1–9]. Among them, caterpillars exhibit a paradigmatic example of non-pedal crawling gait [10], in which—as we will show in the present paper—the competition between body elasticity and adhesion to the substrate plays a key role in maintaining as cyclical the movement triggered by muscle contraction. This kind of locomotion, involving flexible body units that continuously interact with the surrounding space, has attracted increasing interest in the last years, especially in soft robotics aimed at designing deformable structures able to perform several functions in confined and intricate environments [11–13].

Robots employing soft materials in fact provide numerous advantages if compared to conventional ones made by rigid articulating parts, their safety, cost-effectiveness and adaptability [14,15] all contributing to allow automated systems to overcome obstacles and explore areas otherwise difficult to reach [16–19]. Industrial and biomedical applications also exploit highly deformable structures, for instance by designing soft systems inspecting human vessels, for drug delivery and endoscope devices. Therefore, a deep understanding of how biological organisms, at different scales, move through complex habitats is crucial for mimicking their behaviour and for realizing extremely specialized next-generation robots.

A large number of locomotion modalities can be recognized. Among them, how caterpillars move deserves special attention due to the unique crawling mechanism exhibited by these larvae during their movement that call into play the interaction between the intrinsic elasticity of their body segments and prolegs’ grip on the substrate, which allows these larvae to move on different material surfaces, provided that they can adhere on them and regardless horizontal or vertical paths to be faced [20].

Morphological features associated to diverse functions of caterpillars assume in fact a crucial role for their locomotion on several substrates [21] and in achieving anti-predator or anti-parasitoid mechanisms [22–24]. In particular, the caterpillar’s body, which is covered with a periodic shed and a soft cuticle, is segmented into 12–14 subunits, each potentially having prolegs [25]. The abdominal prolegs are mainly involved in locomotion and act like suction cups to attach to—or detach from—surfaces, thoracic legs being used instead for exploring and testing substrates and the environment [25]. Moreover, highly deformable longitudinal muscles along the body can contract to less than 50% or stretch almost double their length [26,27]. Evidence shows that a muscle inelastic contraction, occurring at the terminal part of the animal, activates the forward motion, inducing one proleg at a time to detach from the support, starting from the end segment to the head. Further experimental observations in our lab show that, as the contraction wave advances, the suction cup-like abdominal prolegs surpass their adhesive force and disconnect from the substrate, in this way aiding the lifting phase and allowing the progression of the rear body, thereby guaranteeing steadfast adhesion and effective locomotion.

The vast majority of the literature studies [26,28–30] focuses on mechanical models apt to capture caterpillar locomotion, by employing discrete lumped-mass models subjected to dynamic forces. Most of these research works propose approaches typically adopted in the field of structural dynamics [31–34], thus modelling muscle contraction as a harmonic input and simulating the detachment–attachment cycles of the prolegs as active on/off binary systems. However, while in this manner what one sees is replicated, some underlying mechanisms, such as the interplay between the detachment–attachment of the prolegs and the elastic behaviour of the caterpillar body, could be somehow disregarded or even completely lost, shifting focus of the mathematical modelling of soft invertebrates’ locomotion to the sole active contribution [27,29,35–41].

With the aim of reconstructing the actual mechanism caterpillars employ for locomotion, including the crucial competition between elasticity and adhesion, we provided an essential model, informed by *ad hoc* experimental observations and laboratory tests. We first analysed movements of *in vivo Pieris brassicae* larvae on wooden surfaces, to understand how body’s elasticity interacted with the substrate adhesion regulated by the prolegs during locomotion and attachment–detachment cycles. Also, to obtain some quantitative estimations associated to forces needed for detaching prolegs from wooden surface, a specific experimental setup was conceived, in detailed described below. Then, integrating our laboratory observations and literature evidence [25,42], a one-dimensional discrete model consisting of multiple lumped masses interconnected by springs was adopted to simulate the relative movements and elasticity of each body segment. Additionally, we incorporated a simple support in correspondence of the posterior for replicating the pivot action exerted by the terminal subunit during locomotion, employing Winkler-like constraints to modulate the interplay with the substrate. Detachments of the prolegs from the substrate were thus obtained as a threshold value at the tensile regime of the Winkler-like springs was achieved.

Under the hypothesis of quasi-static movement, thus neglecting inertial and dissipative forces, the crawling motion of the larva is initiated through muscle contractions, modelled as inelastic displacements imposed at terminal pivot, the locomotion resulting from the apparent travelling wave propagating along the whole caterpillar body while involving its elasticity and the interplay with the adhering/de-adhering prolegs cyclically anchoring to the substrate. Numerical tests were also been performed to confirm and validate the proposed approach and to calibrate selected parameters for sensitivity analyses. Finally, the model was enriched by rewriting the equations in a general dynamical framework, analysing the actual influence of inertial and dissipative effects in caterpillar locomotion and discussing the results in comparison with the outcomes from elasto–static assumptions.

Differently from other works, this study may contribute to elucidate—for the first time—how the competition between adhesion and elasticity plays a key role in orchestrating caterpillars’ cyclical and stable movements, suggesting possible new strategies for designing bio-inspired soft robots, capable of moving in confined and limited spaces by exploiting this efficient crawling mechanism that incorporates explicitly the interaction with the substrate.

The paper is organized as follows: §2 shows experimental observations carried out on *in vivo* larvae, starting from the analysis of the morphological characteristics and then paying attention to their locomotion on a wooden substrate; §3 provides the elasto–static modelling of caterpillars’ gait with some insights onto the dynamics conditions, by also validating the proposed mechanical model through numerical experiments; and §4 summarizes the results and reports the final discussions and conclusions.

## 2. Material and methods

To capture the mechanical principles that govern caterpillar locomotion and to achieve a comprehensive understanding of their movement, we preliminarily conducted an experimental campaign in our laboratory that included observations and measurements needed for obtaining qualitative and quantitative information on functional, anatomical and biological characteristics of these larvae and their role in locomotion.

While early literature describes caterpillars’ crawling as legged peristalsis [43,44], recent research studies indicate that they employ a tension-based mechanism founded on the adhesion on arboreal environments [45]. Unlike hydrostatic skeletons, caterpillars rely on a robust grip on the substrate to transmit forces, utilizing their surrounding context as a sustaining structure [25,29,45–47]. On these bases, we investigated the possibility that the locomotion of these larvae was actually allowed by an effective interaction between the muscle contraction that elastically propagates through the soft caterpillar’s body and the adhesion of the prolegs on the support, with attachment–detachment cycles ruled by the continuously changing stress distribution along the body that lead to updating prolegs positions once adhesion force thresholds are reached while a contraction-induced wave travels along the body tracts. Due to the elastic nature of caterpillar tissues and the deformations of the body involved in locomotion, it is also argued that combining elasticity and adhesion is used by caterpillars for moving by maintaining and stabilizing the crawling gait. Also from an evolutionary point of view, facilitating stable elastic movements offers caterpillars advantages such as rapid growth and safe access to distant food sources [25]. This is confirmed by observations, which highlight how these soft-body invertebrates typically exhibit different functional subunits, which can be categorized into the head and thoracic legs, while the posterior part consists of abdominal and a terminal segments, with prolegs predominantly involved in the anterograde locomotion system.

### 2.1. Key insights into locomotion

**Figure 1.**
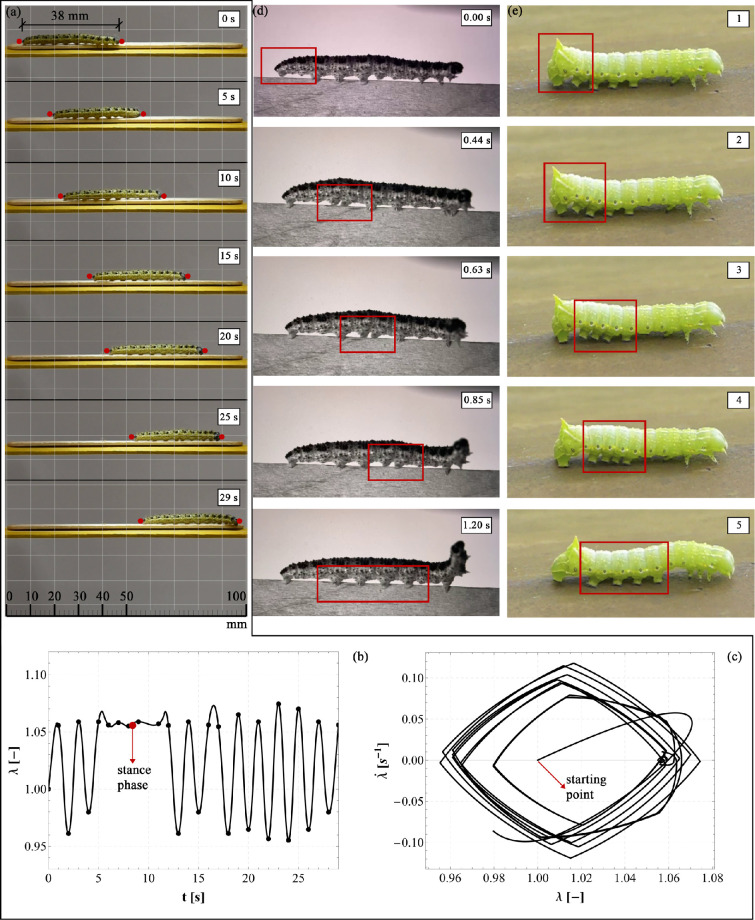
Evidence of caterpillar locomotion: in the left column, part (a), multiple crawling cycles captured by the Nikon D5600 camera are shown over a 29 s interval. During this time, a larva measuring 38 mm in length and weighing 0.35 g travels approximately 50 mm, achieving an average speed of about 1.7 mm s^−1^. Using the red markers in part (a) as reference points for the larva’s current length, the corresponding stretch ratio (i.e. the current length divided by the resting length, $\lambda =L/L_{0}$) is plotted over time and provided in part (b). The stretch history is obtained by interpolating the data points, registered from video analysis via a capture and playback software and marked with a black circle, using a second-order polynomial. A prolonged stance phase is observed approximately from 5 to 11 s. Additionally, the stretch rate, $\dot{\lambda }$, reported in the form of a phase portrait versus λ is provided by numerically deriving over time the stretch history and it is shown in part (c). In the central column, part (d), selected frames from a single crawling cycle (lasting 1.2 s) of a *Pierris brassicae* larva moving along a slender wooden element are presented. These frames, captured by the high-speed camera CMOS MINI FASTCAM AX100, highlight the progression of inelastic contraction as well as the sequential attachment and detachment of the prolegs, marked with red rectangles. Finally, in the right column, part (e), crawling sequences from the video [48] are presented to illustrate the walking behaviour of a different species.

An experimental campaign has been carried out to register the caterpillar motion on horizontal supports, in order to understand the fundamentals of their crawling mechanism to be uploaded as key features into the model for obtaining a biophysics-informed mathematical model. To achieve this goal, the locomotion of caterpillars (specifically, species *Pierris brassicae*) on wooden supports has been recorded using a Nikon d5600 camera and a high-speed Photron FASTCAM Mini AX100 camera (sampling rate of 4000 fps at image resolution of 1024 × 1024 pixels) in some cases where a high number of frames was required in a very short time period to capture relevant behaviours. Two different wooden substrates were considered: the first one is slightly larger than the caterpillar’s body to highlight its cyclic and almost one-dimensional rectilinear motion (figure 1a); the second support is instead made by a slender beam whose width is much lower than the animal’s cross-section size apt to immediately emphasize the subsequent attachments and detachments of the prolegs during a crawl-cycle (figure 1d).

For the purpose of ensuring the repeatability of the experiments, we specify that a birch stick was selected due to its easy availability and fidelity to the natural environment in which caterpillars move. In detail, with reference to its mechanical characteristics, its density varies approximately between 0.60 and 0.75 g cm^−3^, while its Young’s modulus typically ranges from 10 000 to 12 000 MPa and the side Janka hardness is approximately around to 4.0 kN [49].

Interestingly from the laboratory tests, we observed that for the larger supports, the stretch of the entire caterpillar, calculated as the ratio between the current and the resting length of the whole body, varied following a quasi-harmonic trend, as shown in figure 1b,c, the stretch rate values falling approximately into the range [−0.10,0.10].

### 2.2. Role of prolegs and legs in locomotion

**Figure 2.**
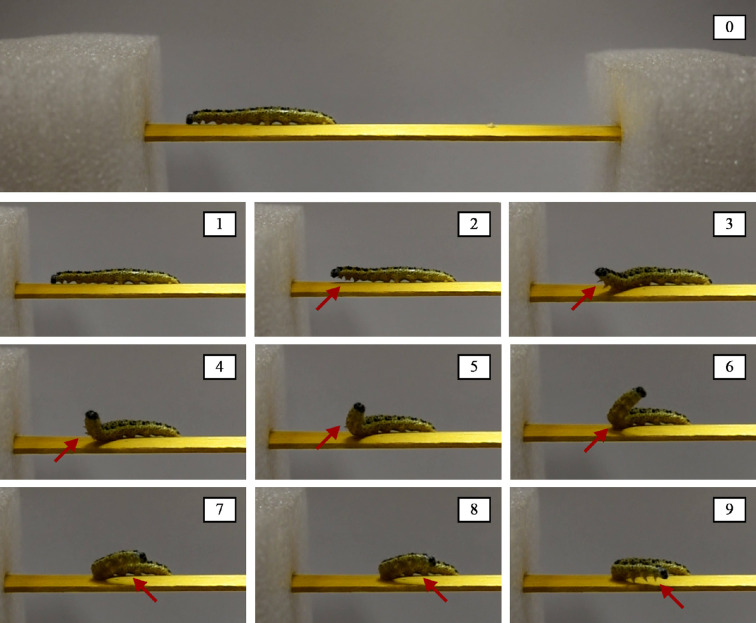
Sequence of snapshots capturing how the caterpillar probes the substrate through its thoracic legs (see the red arrows) before changing its locomotion verse. The images seem to highlight that the animal employs its anterior legs as sensors for testing the nature and the consistency of the substrate before initiating the movement.

The performed experimental tests confirmed the two distinct functions of caterpillars’ abdominal prolegs and anterior thoracic legs somehow mentioned in [25], highlighting how the formers are mainly engaged during locomotion, while the latters are employed for testing/inspecting the substrata before initiating the movement. We can also see that—by observing snapshots of figure 1d—the terminal body segment acts as a pivot point on the substrate and allows the propagation of the muscle contraction to other subunits modulating the locomotion thanks to the adhesion of the abdominal prolegs. As emphasized by the red contours in figure 1d,e, prolegs on a single body segment are lifted progressively during this crawling motion, in parallel to the diffusion of the muscle contraction. In detail, each proleg goes through two phases during each crawl: the swing phase during which the proleg is moved forward, and the stance phase, during which the proleg is in contact with the ground or the support. It is interesting to notice that—as highlighted by the red rectangles in figure 1d,e—when the muscle contraction has reached the i-th body unit and has overcome the adhesion level of the prolegs located in those segments, detachment occurs, also gaining a further advantage from the pull-off phenomenon for continuing and stabilizing locomotion. Additionally, experimental evidence revealed that caterpillars analyse the consistency of the substrate using their anterior thoracic legs, subsequently adjusting the gripping of their abdominal prolegs to facilitate movement on the surface. As one can observe from the snapshots collected in figure 2, the larva adheres with the medium-rear part of its body to the substrate and bends its anterior segments to explore the environment before initiating the movement and changing its motion verse.

### 2.3. Role of adhesion and measurements

**Figure 3.**
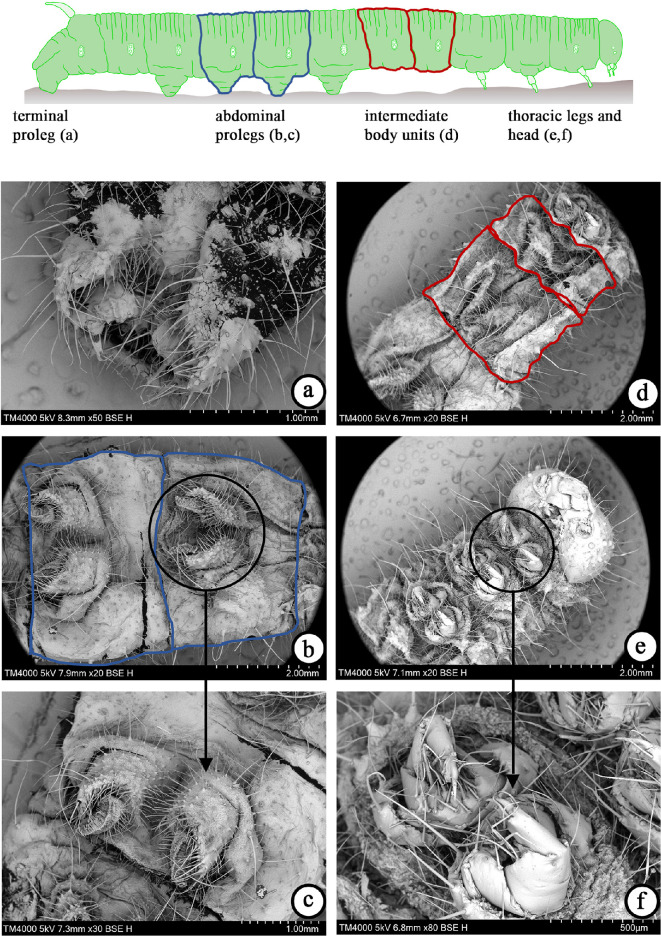
Morphological characteristics of the several subunits of caterpillars (*Pierris brassicae* species) observed through SEM: (a) terminal segment with the prolegs captured in the retracted phase (scale bar 1 mm), (b) abdominal segments (scale bar 2 mm) with a zoom, (c) highlighting the microstructures that act as section-cups and modulate adhesion on the substrate (scale bar 1 mm), (d) intermediate body units (scale bar 2 mm), (e) thoracic legs and head (scale bar 2 mm) with (f) an enlargement (scale bar 0.5 mm) focusing on the hooks-like legs probably adopting for testing the support before initiating locomotion. This figure synoptically collects the details of the anatomically different parts of the larva, each one corresponding to a specific function, as also specified in the dedicated literature [25,50–52].

To gain further insights into the morphological features of the two kinds of prolegs/legs, observations through the scanning electron microscope (SEM) Hitachi TM4000 II were performed and the results illustrated in figure 3. Several images were provided, with a special focus on the body’s parts that play a key role in the activation and propagation of the locomotion.

**Figure 4.**
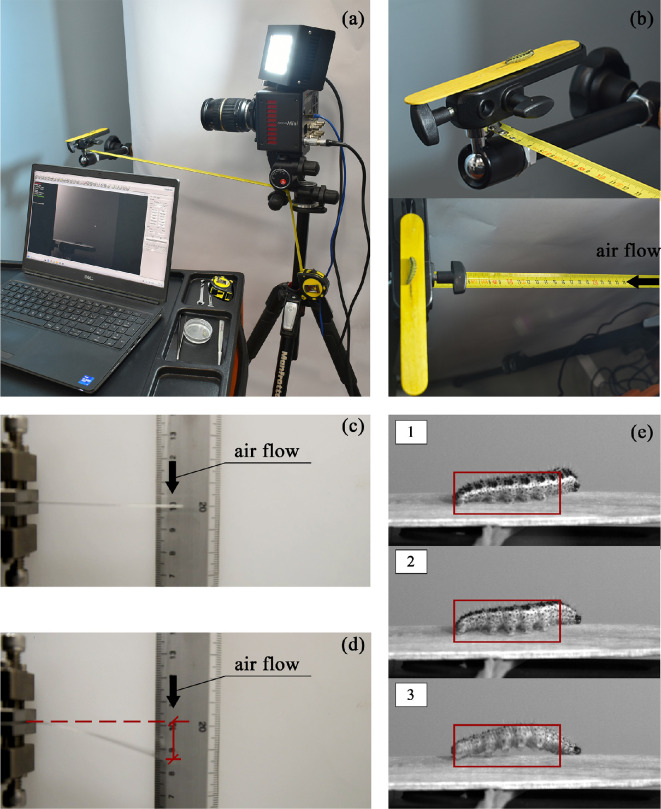
Measuring adhesion in caterpillar's abdominal prolegs: (a) experimental setup with high-speed camera registering the adhesion/detachment phases of the caterpillar on a wooden support; (b) details of the support and the ruler interposed between the camera and the substrate (the black arrow indicates the air flow ejected from the compressor that causes the caterpillar's detachment at 105 mm); (c, d) calibration and measurement of the force exerted by the air compressor, by calculating the deflection equal to 14 mm of a clamped steel beam (dimensions 62.5 × 4.43 × 0.25 mm and Young’s Modulus 210 × 10^3^ MPa); (e) snapshots captured by the camera of three states: in the first one, no air flow is applied and the caterpillar perfectly adheres to the surface, the second one reproduce an intermediate situation where an air force of 0.10 N loaded the prolegs, the third one is the frame where detachment occurs corresponding at a force of 0.20 N.

In particular, the SEM observations corroborated the findings reported in [25,50–52]. Indeed, the image allows to recognize the diverse morphologies of each segment of the caterpillar that call into play distinct functions, some of them activated during locomotion. In figure 3, it can be clearly distinguished these larva body parts: the anterior one, characterized by three pairs of thoracic legs that possess an exoskeleton and are not strictly involved in the crawling gait, and the prolegs, located at the abdominal subunits and mainly engaged during locomotion. By combining the SEM images and the experimental observations mentioned above, some relevant conclusions can be drawn for the model. The thoracic legs (figure 3e,f) look morphologically like hooks and actually work as sensors to probe the substrate, while the abdominal prolegs (figure 3b,c) have the shape of suction cups and are hence utilized during each crawl-step of the movement for adhering to or detaching from the surfaces. Importantly, figure 3a confirms, from the morphological standpoint, that the terminal segment of the caterpillar where the prolegs, usually referred to as ‘claspers’ [25], acts as pivot point for initiating the locomotion. Since experimental tests performed also showed us that both the stiffness of the caterpillar’s body and the adhesion of the abdominal prolegs play equally important roles for the locomotion mechanism, the estimation of these two parameters being of paramount importance for calibrating the mechanical model. We started considering that, in the inherent literature, the stiffness of the caterpillar’s musculature was already estimated; as an example, the reference [26] is very useful to this scope. However, to the best authors’ knowledge, it appears that no direct measured values were available for the adhesion forces needed to detach caterpillar prolegs from the surface where they anchor. To address this specific point, a dedicated experimental setup to provide an estimation of the threshold adhesion value was prepared and observations were made (figure 4). In detail, the high-speed camera Photron CMOS MINI FASTCAM AX100 was positioned in front of a support holding the wooden substrate to which a caterpillar of the species *P. brassicae* adhered, in order to record, via a work station, the detachment of the caterpillar (figure 4a). Additionally, a ruler was interposed between the camera and the support, which was necessary to modulate the pressure exerted on the caterpillar through an air compressor. In fact, by iterating the test multiple times, starting from the camera and progressively approaching the support where the caterpillar adhered, the minimum distance from which air was blown to induce detachment was measured (figure 4a,b). After recording this distance, we calculated the effective force exerted on the caterpillar, by evaluating the deflection of a clamped thin steel beam, whose mechanical and geometric properties were known, and then estimating the applied force following an inverse method (figure 4c,d). In particular, the total force (scaling as $∼E\;I\;\delta \;L^{-3}$, L being the beam length, E the Young’s modulus, I the moment of inertia of the beam cross-section and δ the measured deflection at the beam end) was then divided by the four pairs of prolegs present on the abdominal segments of the caterpillar. From these calculations, the adhesion force acting on each subunit and producing detachment was estimated as approximately 0.20/4=0.05N.

## 3. Mechanical model

### 3.1. Elasto-static modelling of caterpillars’ gait cycles

A one-dimensional discrete lumped mass system has been employed for tracing back the caterpillar locomotion. In detail, the physical-based model reported in what follows has purposely a few parameters, in order to reproduce the crucial aspects of the larva’s crawling gait.

**Figure 5.**
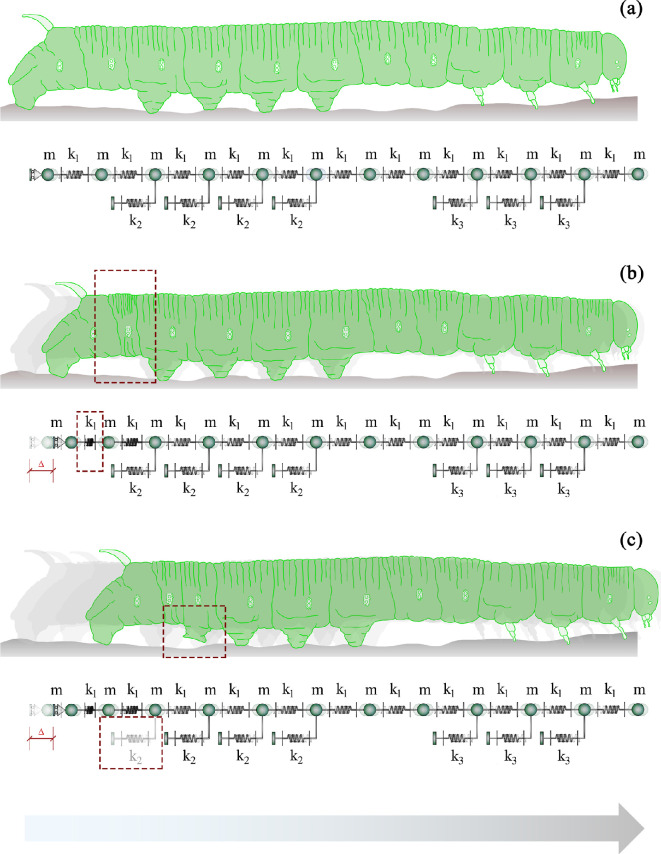
Graphical representation showing the subsequent phases of the caterpillar's crawling gait: (a) resting phase, (b) activation of the locomotion through the inelastic contraction driven by the terminal proleg, (c) detachment of the first proleg when the threshold is achieved before the elastic repartition of the stress into the subsequent mechanical configuration. The direction and the verse of the motion is highlighted by the grey arrow. The parameter m refers to the mass of the subunits, while $k_{1}$, $k_{2}$ and $k_{3}$ correspond to the stiffness of the body segments, the abdominal prolegs and the thoracic legs, respectively.

Since the inertial and dissipative effects can be initially ignored for describing the key features of locomotion, an elasto-static approach was first assumed. The caterpillar’s body segments are represented by lumped masses connected by springs in series that reproduce the elasticity of the animal’s cuticle and the interaction crawler-substrate is modulated by means of horizontal Winkler-like springs that lie in correspondence to the prolegs (see figure 5). Winkler springs are often used in mechanics, as well as generally in the engineering realm, to represent how an elastic substrate behaves under loads and interacts with the upper structure [53,54]. This elastic interaction is idealized as a series of independent springs. Each spring supports a specific point on the structure and responds individually to the applied load at that point. The key assumption is that the reaction force at any given point depends only on the displacement at that position and—by adopting a linearly elastic constitutive law—is proportional to it. This means the springs are not interconnected, and there is no transversal force transfer among them. Given the nature of the abdominal prolegs on the segmented caterpillar body, this model can perfectly match both the mechanical and discrete morphological structure of the larva.

Additionally, as already mentioned above, a support was placed on the terminal lumped mass, for reproducing the final segment of the caterpillar acting as a pivot point that serves as a trigger of the locomotion, consistently with the experimental observations of the actual locomotion modes.

**Figure 6.**
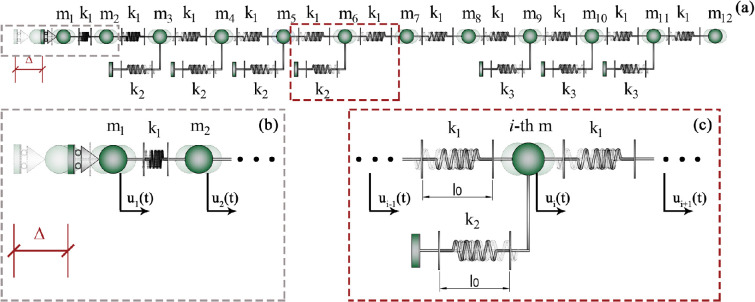
Discrete lumped masses system adopted for modelling caterpillars' locomotion (a), where $k_{1}$ is the inter-masses springs' stiffness, while $k_{2}$ and $k_{3}$ are the Winkler-like springs' stiffness referring respectively to abdominal and thoracic legs; $l_{0}$ is the resting length and $m_{i}$ the i-th mass, representing the body segments of the caterpillar. The gray inset (b) provides detail on the posterior subunits, being the simple support modelling the terminal prolegs acting as pivot point with a prescribed displacement Δ and the red rectangle (c) focuses on the i-th mass.

Linear elasticity was considered for inter-masses and Winkler-like horizontal springs, the latter with a threshold $u_{y}$ at a tensile limit for simulating the detachment of the prolegs from the substrate. Standard methods of theoretical and applied mechanics [55] were thus applied to solve the structural one-dimensional model with a prescribed displacement at the end. As reported in figure 6, the system’s coordinates are the displacements of the lumped masses (say $u_{i}(t)$), with the sole exception of the one corresponding to the terminal proleg, $u_{1}(t)$, whose displacement is imposed as contraction stimulus and set to Δ.

The total elastic potential energy of the system, here appointed as $U_{\text{EL}}$, can be given explicitly as follows:


(3.1) $$\begin{array}{rl} & U_{\text{EL}}=\;\;\frac{1}{2}\sum_{i=2}^{n-1}k_{2}[i]\left(u_{i}(t)\right){,}^{2}+\frac{1}{2}k_{1}\sum_{i=2}^{n-1}\left(u_{i+1}(t)-u_{i}(t)\right){,}^{2}+\\ & \;\;\;+\frac{1}{2}k_{1}\left(u_{2}(t)-\Delta \right){,}^{2},\;\;n=12,\;\\ & \text{where}\;\;k_{2}[i]=\left\{ \begin{array}{ll} & \alpha \;k_{1}\;\;\text{if}\;\;i=\{3,4,5,6\}\;\;\text{with}\;\;u_{i}\leq u_{y}\\ & 0\;\;\;\text{otherwise} \end{array}\right., \end{array}$$

being α the ratio between the stiffness of Winkler-like and inter-masses springs, i.e. $k_{2}/k_{1}$. In order to generalize the expression in the dynamic phase disclosed in the subsequent section, the displacements $u_{i}(t)$ are reported as time-dependent unknowns in equation (3.1); however, by neglecting both inertial and dissipative effects at this elasto-static stage, the time variable t is not explicitly considered. Since, as experimentally validated in §2, thoracic legs with stiffness $k_{3}$ (see figure 6) play a minor role in crawling locomotion, their contribution was neglected in equation (3.1).

By minimizing the total elastic potential with respect to the unknown displacements from the $2^{\text{nd}}$ to the $12^{\text{th}}$ DoF, linear equilibrium equations were derived. With reference to the i-th inter-mass spring (figure 6b), the i-th equation can be written as:


(3.2) $$\begin{array}{lcr} & k_{1}(u_{i}-u_{i-1})+k_{2}[i]\;(u_{i})-k_{1}(u_{i+1}-u_{i})=0. & \end{array}$$

By activating the prescribed displacement $u_{1}(t)=\Delta$, the analytical solutions of the n−1 equation (3.2) have the following form:


(3.3) $$\begin{array}{rl} & u_{2}=\frac{\alpha^{4}+7\alpha^{3}+15\alpha^{2}+10\alpha +1}{D}\Delta \\ & u_{3}=\frac{\alpha^{3}+5\alpha^{2}+6\alpha +1}{D}\Delta \\ & u_{4}=\frac{\alpha^{2}+3\alpha +1}{D}\Delta \\ & u_{5}=\frac{\alpha +1}{D}\Delta \\ & u_{6}=u_{7}=u_{8}=u_{9}=u_{10}=u_{11}=u_{12}=\frac{\Delta }{D},\\ & \text{where}\;\;\;D=2\alpha^{4}+13\alpha^{3}+25\alpha^{2}+14\alpha +1\;. \end{array}$$

It is worth underlining that—for any values of the stiffness ratio α in an interval that makes sense from the physical standpoint, i.e. α>0—the displacements decrease when moving away from the inelastic Δ activated for triggering the locomotion.

**Figure 7.**
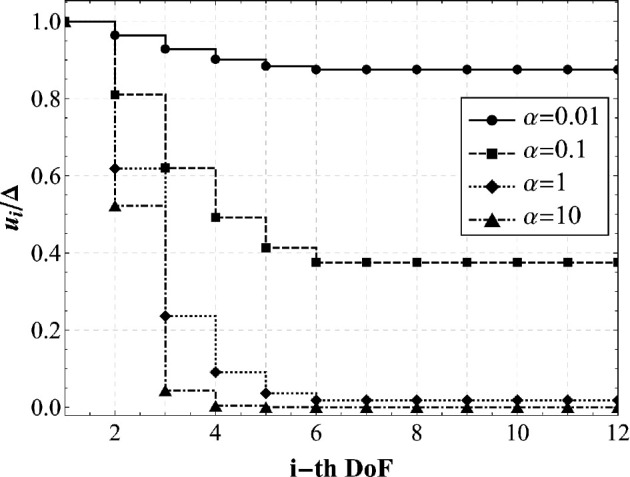
Effects of the variation of the stiffness ratio α, on the displacements solutions of each DoF (equation (3.3)), normalized with respect to Δ. The parameters are set as $\Delta ≃1.45\times 10^{-3}\;m,\;l_{0}=2.0\times 10^{-3}\;m,\;\alpha =0.01,\;u_{y}=l_{0}/1.5=1.33\times 10^{-3}\;m,\;k_{1}=5\;N\;m^{-1}$. While the geometric and the mass parameters have been experimentally measured, the stiffness $k_{1}$ has been evaluated starting from the findings of the research paper [26], where the Young’s modulus of the caterpillar muscle tissue is estimated approximately 2.5kPa..

To thoroughly investigate the system’s behaviour, by fixing Δ, the variation of the stiffness ratio strongly affects the displacements’ distribution of each DoF, as depicted in figure 7. Therefore, it is possible to gain an immediate understanding, both qualitatively and quantitatively, of the influence of the α parameter on the model, by visualizing figure 7: one can observe that the displacement curves tend to flatten when the stiffness ratio decreases. As expected, the displacement is always maximum near the inelastic distortion applied to the end of the caterpillar, and attenuates in magnitude for the other masses.

**Figure 8.**
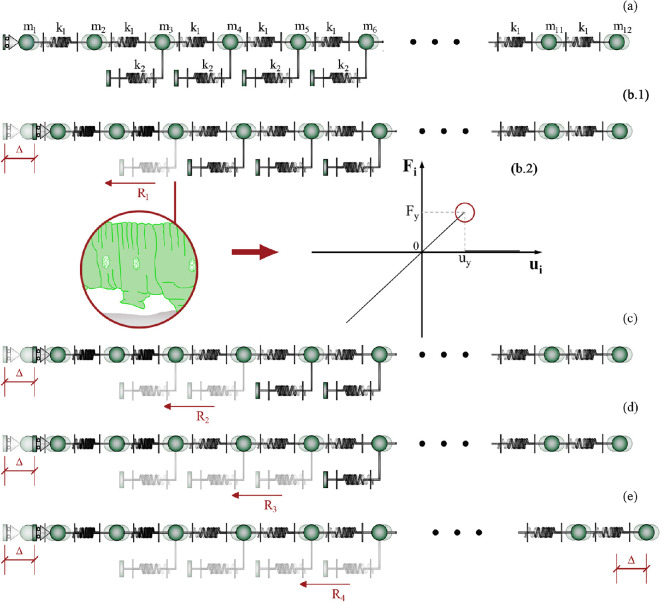
Illustration reproducing the substeps that occur during caterpillar locomotion with the progressive detachment of prolegs: the stance phase is depicted in configuration (a), while configuration (b.1) reports the substep 1, where the loss of adhesion of the Winkler-like spring is achieved at the threshold $u_{y}$ provided in inset (b.2); part (c), (d) and (e) refer to the subsequent substeps where the two adjacent constraints that modulate the substrate interaction attain the detachment force. This gait continues up to the last proleg is detached, by completing one crawl-cycle and by leading the whole body to move of Δ (substep 4).

Starting from the first proleg encountered immediately after the inelastic contraction that activates locomotion, the anchors are detached one at a time during each crawling sub-step. This sequential detachment allows the caterpillar to release its constraints on the ground and move forward, as schematically illustrated in figure 8. The attachment and detachment cycle of prolegs were mathematically reproduced by introducing a threshold on the adhesion energy of the Winkler-like springs that modulate the interaction with the substrate (see figure 8b.2). In particular, when the threshold force of the spring, say $F_{y}$, is achieved in the tensile regime, the constraint spring is turned off and the muscle contraction propels forward the animal through the traveling of the inelastic deformation in the actual deformed configuration. It is worth specifying that, after completing the crawl-cycle, all Winkler-like springs are restored, thus simulating the active attachment of the caterpillar’s prolegs.

From the mechanical standpoint, beyond the stiffness ratio, the calibration of the inelastic displacement initiating locomotion is crucial to ensure successful locomotion of the larva. Specifically, this parameter must be calibrated to match the value required to reach the detachment threshold for at least one of the prolegs across all subsequent configurations caused by the progressive loss of anchoring points. This process culminates in the final configuration, where only the last suction cup remains attached before finally enabling the rigid translation of the entire caterpillar body, as illustrated in figure 8e. Therefore, by fixing the mechanical characteristics of the caterpillar, the value of the inelastic contraction that can allow locomotion, denoted as $\tilde{\Delta }$, can be evaluated as the maximum of $\left\{\Delta_{1},\Delta_{2},\Delta_{3},\Delta_{4}\right\}$, it representing, at the *i*-th sub-step, the minimum inelastic displacement that the terminal subunit must generate to detach at least one proleg in the corresponding *i*-th configuration.

In detail, it is possible to analytically prove that the maximum reaction force, $R_{i}$, is localized at the first step in the spring immediately after the imposed displacement; at the *i*-th substep (i>1), the maximum stress is registered at the *i*-th Winkler-like constraint. In what follows, from the final segment of the caterpillar to the head part, the values of $\Delta_{i}$ such that the threshold $u_{y}$ is reached for at least one spring can be written as:


(3.4) $$\begin{array}{rl} & \Delta_{1}=u_{y}\frac{2\alpha^{4}+13\alpha^{3}+25\alpha^{2}+14\alpha +1}{\alpha^{3}+5\alpha^{2}+6\alpha +1}\\ & \Delta_{2}=u_{y}\frac{3\alpha^{3}+13\alpha^{2}+12\alpha +1}{\alpha^{2}+3\alpha +1}\\ & \Delta_{3}=u_{y}\frac{4\alpha^{2}+9\alpha +1}{\alpha +1}\\ & \Delta_{4}=u_{y}(5\alpha +1),\\ & \text{with}\;\;\Delta_{i}\leq l_{0}. \end{array}$$

The displacement $\tilde{\Delta }$ required for completing the crawl-cycle is strongly dependent on the stiffness ratio parameter and the position of the prolegs. Additionally, it is worth stressing that the compatibility of the $\Delta_{i}$ values in (3.4) should be verified by checking that each value is lower or at least equal to the resting length $l_{0}$ of inter-mass springs. This inequality represents *de facto* a constraint condition that avoids interpenetration between the terminal subunit and the adjacent mass.

By numerically comparing $\Delta_{i}$ of equation (3.4) each other, one may notice that, for the vast majority of α values, the curves representing $\Delta_{3}/u_{y}$ and $\Delta_{4}/u_{y}$ are always upper bounds of the other ones, thus emphasizing that, generally, the gripping in the anterior abdominal prolegs of the caterpillar is mainly responsible of the intensity of the inelastic displacement to be prescribed to the system for ensuring the progressive detachment of all anchors, so allowing the locomotion. More in detail, it is possible to make explicit $\tilde{\Delta }$ as a function of α and $u_{y}$:


(3.5) $$\begin{array}{rl} & \tilde{\Delta }≃\left\{ \begin{array}{ll} \Delta_{2}, & \;\text{ if }0<\alpha \leq 0.130\\ \Delta_{3}, & \;\text{ if }0.130<\alpha \leq 3\\ \Delta_{4}, & \;\text{ if }\alpha >3 \end{array}\right.,\\ & \text{with}\;\;\tilde{\Delta }\leq l_{0}. \end{array}$$

It is worth highlighting again that we indicate with $\tilde{\Delta }$ the value of the inelastic contraction—appointed generically as Δ previously—that allows the caterpillar to detach progressively all its prolegs, thus overcoming for all segments the threshold displacement. In equation (3.5), the approximation is related to the inequalities in terms of α.

**Figure 9.**
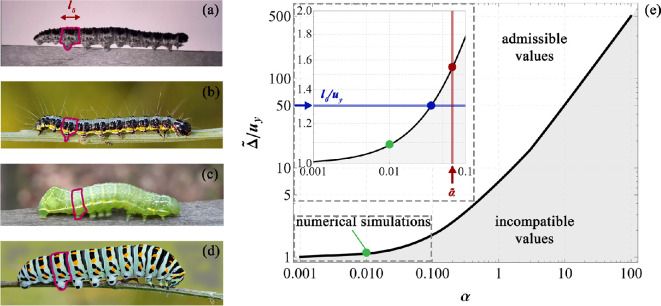
A qualitative comparison of the morphological features of some caterpillar species, i.e. *Pierris brassicae* (a), *Uresiphita gilvata* (b), *Amphipyra pyramidea* (c) and *Papilio machaon* (d) is provided, recalling how variations in geometric parameters and body/prolegs elasticity can influence gait triggering action. To highlight this, part (e) reports a region plot in logarithmic scale analysing the possible combinations of parameters of the linear system, by highlighting when locomotion is made possible: the dimensionless ratio $\tilde{\Delta }/u_{y}$ is provided as a function of the stiffness ratio α. The inset in part (e) reports a zoom on the range of low stiffness ratios. In detail, the $\tilde{\Delta }/u_{y}$ parameter represents the ratio between the inelastic contraction to be applied for triggering locomotion and the threshold displacement for detaching prolegs, which somehow quantifies the energy needed to activate and to maintain locomotion with respect to the elastic energy cumulated in the Winkler-like springs before detaching from the substrate. The grey area refers to incompatible values, where interpenetration between adjacent masses occurs, while the admissible states are included in the complementary zone. By recalling that $\tilde{\Delta }$ must be $\leq l_{0}$ for avoiding interpenetration between the first and the subsequent body segment, one can fix—as a limit case—the ratio $l_{0}/u_{y}$ depending on the analysed caterpillar species, and *a priori* evaluate the optimal stiffness ratio value that minimizes the input energy of the larva for achieving and maintaining locomotion (see the blue circle on the black curve). The dimensionless parameter $l_{0}/u_{y}$ characterises the relation between the resting length of the body units and the displacement threshold of the Winkler-like constraints, thereby defining a geometric ratio that may play a crucial role in the evolutionary dynamics of caterpillars. Moreover, by selecting a prescribed stiffness ratio, we can predict the minimum inelastic displacement $\tilde{\Delta }$ that initiates and maintains stable the locomotion (see the red circle on the black curve). The point related to the numerical simulations illustrated in this work is marked with a green circle.

Therefore, by *a priori* setting the stiffness ratio parameter α and the threshold $u_{y}$, the proposed model is capable to theoretically predict the displacement $\tilde{\Delta }$ to be assigned to the terminal subunit for triggering and maintaining the locomotion for the entire crawl-cycle. However, to ensure geometric compatibility of the overall system, it must be recalled that the prescribed $\tilde{\Delta }$ has to be lower than the resting length $l_{0}$ of the inter-masses springs at all subsequent sub-steps, thus avoiding interpenetration between adjacent segments. This fully parametric investigation is anticipated to offer a model that enhances the biomechanical understanding of caterpillars, providing insights for the design and development of bio-inspired devices that replicate their gait. By estimating the stiffness ratio parameter α—which is determined by the resting lengths and elasticity of body segments and prolegs—its influence on the moving mechanism of various species (for instance, see figure 9a–d) can be studied. Essentially, the proposed model could serve as a robust tool for analysing caterpillar locomotion in relation to their mechanical properties and, more significantly, for predicting the movement patterns of *ad hoc* designed prototypes inspired by the biomechanics of these *larvae*.

Moreover, as previously mentioned, the inelastic displacement that initiates locomotion can be expressed as a function of the stiffness ratio α (refer to the plot in figure 9e). However, the additional constraint inequality ($\tilde{\Delta }\leq l_{0}$)—introduced to prevent interpenetration between adjacent masses—divides the plot into two distinct domains: one representing admissible values, the other region corresponding to geometrically incompatible states, where overlapping between adjacent segments occurs. Consequently, this plot links the geometric and elastic parameters of caterpillars, emphasizing the intrinsic connection between their biological/morphological features and mechanical properties. Therefore, this figure 9e aims to highlight how, from a mechanical point of view, capturing the differences between species could be highly valuable. For biologists, it offers a pathway to understand morphological differences among caterpillars based on locomotion outcomes. Meanwhile, engineers and scholars dealing with bio-inspired design may find such insights useful when considering different species and tailoring designs to meet diverse needs associated to different functional scenarios. In fact, figure 9 assumes a twofold purpose: on the one hand, it acts as a consistency check for the mechanical analysis based on the proposed model; on the other hand, it can be used as a design chart to determine the stiffness ratio or to calibrate the inelastic contraction to be imposed on the system in potential bio-inspired prototypes for soft robotics applications mimicking caterpillar locomotion.

By evaluating $\tilde{\Delta }$ as expressed in (3.5), the illustrated step-by-step strategy has been implemented into the symbolic and numerical environment of Mathematica [56] for faithfully capturing the motion of the larva, by following the algorithm reported in the following:


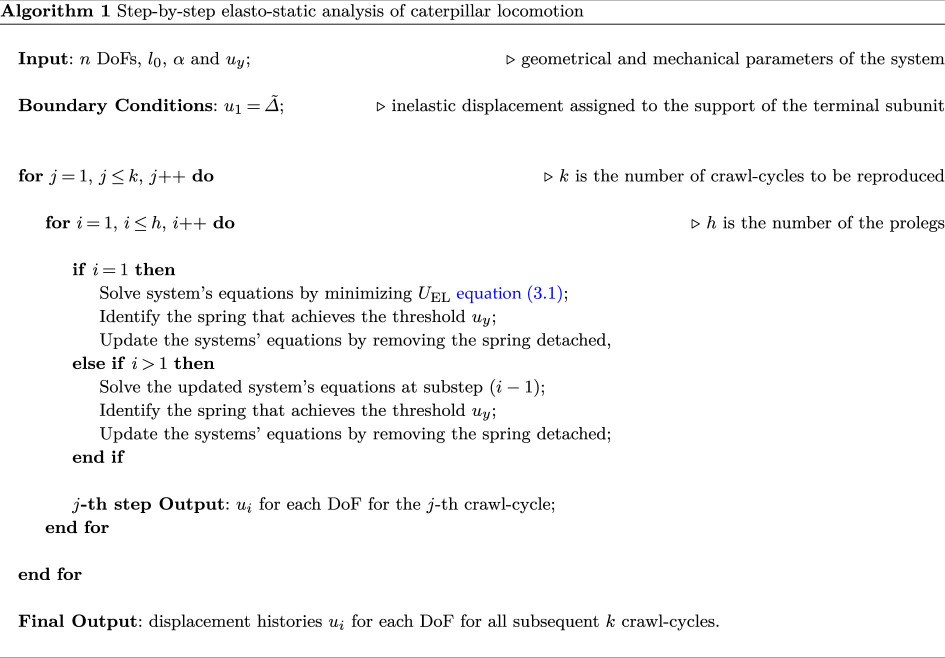


**Figure 10.**
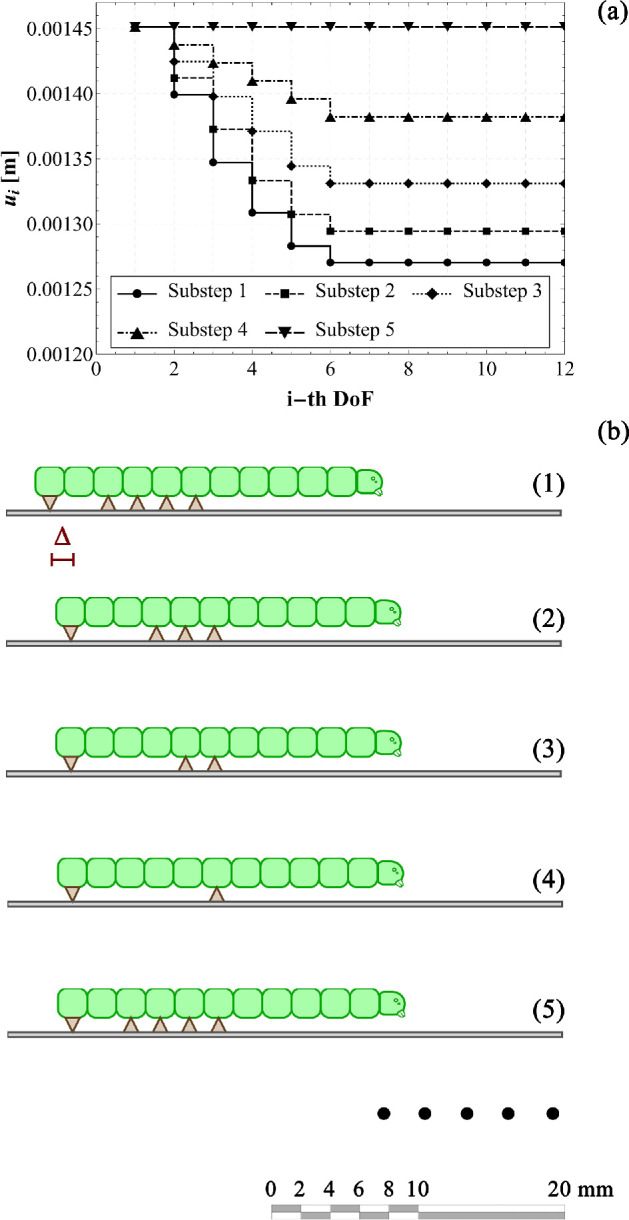
Numerical example of the elasto-static solutions in the linear elasticity framework: displacement values $u_{i}$ as a function of the i-th DoF at all substeps (a) and a graphical representation of them by highlighting the attachment/detachment of the prolegs depicted through brown triangles (b). At the fifth step, the caterpillar achieves its final position, thus translating each DoF of $\tilde{\Delta }≃1.45\times 10^{-3}\;m,\;\text{being instead}\;l_{0}=2.0\times 10^{-3}\;m,\;\alpha =0.01,\;u_{y}=l_{0}/1.5=1.33\times 10^{-3}\;m,\;\text{and }k_{1}=5\;N\;m^{-1}$.

**Figure 11.**
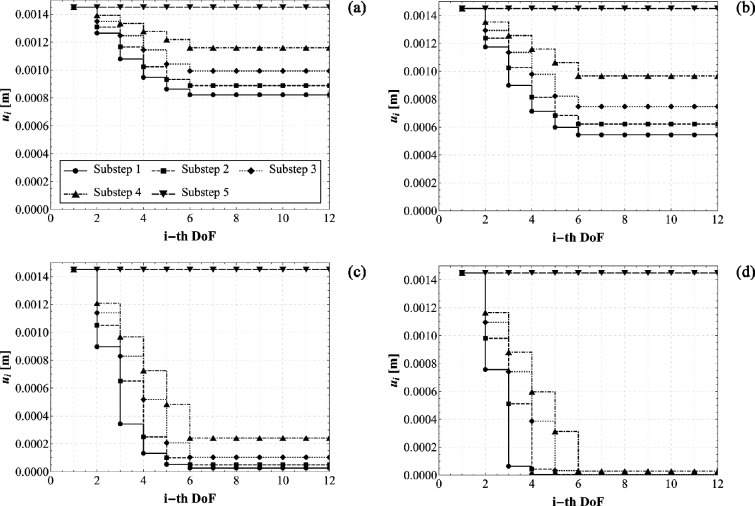
Elasto-static solutions of the caterpillar locomotion varying α: displacement values $u_{i}$ as a function of the i-th DoF at all substeps. For all the plots these parameters are fixed: $\tilde{\Delta }≃1.45\times 10^{-3}\;m,\;l_{0}=2.0\times 10^{-3}\;m\;\text{and}\;k_{1}=5\;N\;m^{-1}$. The displacement threshold $u_{y}$ is modulated in order to keep invariant the triggering $\tilde{\Delta }$ calculated as made explicit in (3.5), therefore, the following parameters are assumed: $\alpha =0.05,\;u_{y}≃1.0e-3\;m\;(\text{a}),\;\alpha =0.1,\;u_{y}≃8.1e-4\;m\;(\text{b}),\;\alpha =1,\;u_{y}≃2.1e-4\;m\;(\text{c}),\;\alpha =10,\;u_{y}≃2.8e-5\;m\;(\text{d})$.

By implementing this step-by-step procedure, the displacement history can be made explicit and the elasto-static problem previously presented can be solved. Figure 10 shows—as an example—the displacement distribution at each sub-step for a selected set of parameters of engineering interest. From the numerical set of parameters, the order of magnitude of the internal actions and reaction forces is approximately 10mN. However, it is worth remarking that, thanks to the fully parametric approach here proposed, we are able—at least in principle—to trace back the gait of several species of caterpillar, by varying their stiffness, adhesion, number of body units and position of the prolegs. To demonstrate the capabilities of the proposed model, we present a sensitivity analysis in which the displacements of each degree of freedom are evaluated for different values of the stiffness ratio α (specifically, 0.05, 0.10, 1 and 10). These values range from very soft prolegs with respect to the caterpillar body to very stiff ones, while all other system parameters were held invariant, including the resting length of the springs and the body’s stiffness. From the plots shown in figure 11, two key observations can be made: first, at each sub-step, increasing the stiffness ratio results in a greater displacement difference between adjacent segments; second, for lower α values, the transitions between subsequent sub-steps appear smoother and more prone to be uniform. For the sake of brevity, only the results concerning variations in the stiffness ratio are presented. This choice is motivated by the practical utility of estimating this parameter *a posteriori*, once body stiffness, number of segments and position and number of abdominal prolegs are known. The stiffness ratio α is particularly challenging to estimate directly, as assessing the stiffness of suction cup-like prolegs can be inherently difficult. Figure 11 seems to suggest that lower stiffness ratios may enhance the stability and periodicity of locomotion during each sub-step.

### 3.2. Dynamic modelling: validation of the elasto-static assumptions

**Figure 12.**
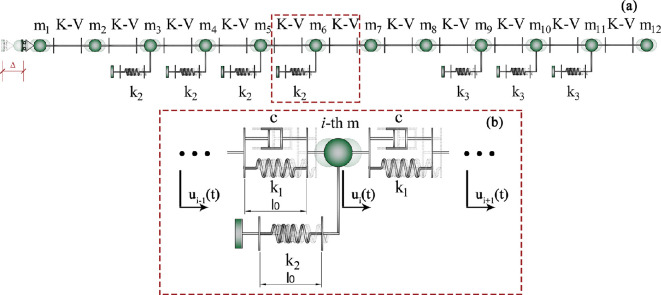
Discrete dynamic lumped masses model adopted for modelling caterpillar locomotion and accounting for the viscosity of the cuticle/muscle tissue through linear dashpots, whose damping coefficient is appointed as c, placed in parallel to inter-masses springs, thus obtaining several Kelvin-Voigt (K-V) links in series (b). Winkler-like springs are still considered for modulating the caterpillar-substrate interaction.

With the aim of assessing the effects of the inertial and dissipative forces previously disregarded, a dynamic model was here considered. Indeed, the system’s dynamics allows an accurate and more realistic examination of the caterpillar’s locomotion and a better understanding of the reliability of the quasi-static assumptions introduced in the essentail model discussed above. Therefore, the final scope of this subsection is to evaluate if the dynamics can significantly affect the overall system’s response, by assuming—in the numerical model proposed in what follows—the set of parameters derived from the literature and preliminary estimated through experiments. To account for the viscosity of the caterpillar’s cuticle, a dashpot was thus placed between all adjacent lumped masses, in parallel with respect to the springs, thus configuring standard Kelvin-Voigt (K-V) links arranged in series, as depicted in figure 12. This upgrade can be particularly interesting for biological tissues, which, as well-known, often exhibit viscoelastic behaviour, which in some cases can significantly influence their response to loads and resulting dynamic stress distributions.

The explicit dynamics of the system also allowed us to investigate the transient regime experienced by each body segment during the caterpillar’s locomotion. To do this, we evaluated the kinetic energy, named T, and the dissipation term, say D, under the hypothesis of linear damping as follows:


(3.6) $$\begin{array}{lcr} & \begin{array}{rl} & T=\sum_{i=1}^{n}\frac{1}{2}m({\dot{u}}_{i}(t){)}^{2},\\ & D=\sum_{i=1}^{n}\frac{1}{2}c({\dot{u}}_{i+1}(t)-{\dot{u}}_{i}(t){)}^{2}, \end{array} & \end{array}$$

where c is the damping coefficient and symbol ‘dot’, as usual, refers to time derivative of the displacement functions.

Standard approaches in analytical mechanics allow us to derive the dynamics equations. By introducing the Lagrangian Functional $L(u(t),\dot{u}(t),t)$ as the difference between the kinetic (3.6) and the elastic potential energy (3.1), i.e. $T-U_{\text{EL}}$, the Euler-Lagrange equations can be written as follows:


(3.7) $$\begin{array}{rl} & \frac{\partial L}{\partial u_{i}}(u(t),\dot{u}(t),t)-\frac{d}{dt}\frac{\partial L}{\partial {\dot{u}}_{i}}(u(t),\dot{u}(t),t)=\\ & \;=\frac{\partial D}{\partial {\dot{u}}_{i}}(u(t),\dot{u}(t),t)\;\forall i\in \{1,\cdots ,n\}, \end{array}$$

being the displacements of each lumped mass the unknowns of the system, with the sole exception of $u_{1}(t)$ that is set equal to $\tilde{\Delta }$.

With reference to the *i*-th lumped mass figure 12b, by still assuming $k_{3}$ equal to 0, the i-th time-varying differential equation takes the following form:


(3.8) $$\begin{array}{rl} & k_{1}(u_{i}(t)-u_{i-1}(t))+\alpha \;k_{1}(u_{i}(t))-k_{1}(u_{i+1}(t)-u_{i}(t))+\\ & \;+m\;{\ddot{u}}_{i}(t)=-2\;\xi \sqrt{k_{1}\;m}\;({\dot{u}}_{i}(t)-{\dot{u}}_{i-1}(t))+\\ & \;+2\xi \sqrt{k_{1}\;m}\;({\dot{u}}_{i+1}(t)-{\dot{u}}_{i}(t)), \end{array}$$

where the damping coefficient c can be calculated as $2\;\xi \sqrt{k_{1}\;m}$, being ξ the dimensionless damping ratio. It should be stressed that, also in the dynamic model, the term $\alpha \;k_{1}(u_{i})$ turns off when the threshold $u_{y}$ is exceeded.

**Figure 13.**
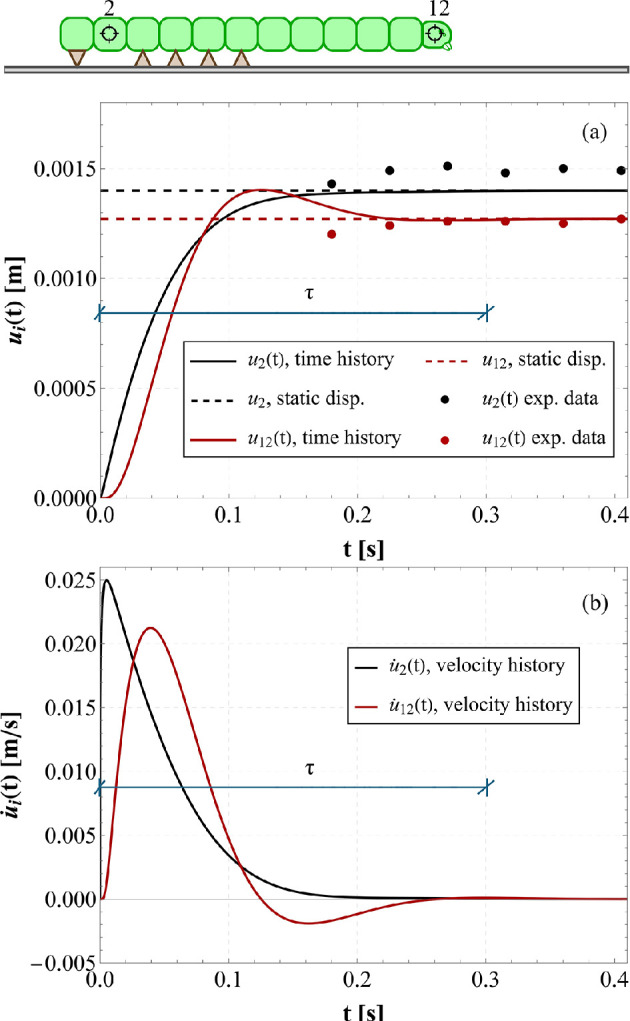
By assuming the following set of parameters $m=1\times 10^{-4}\;kg,\;l_{0}=2\times 10^{-3}\;m,\;u_{y}=1.33\times 10^{-3}\;m,\;k_{1}=5\;Nm^{-1},\;\alpha =0.01,\;\tilde{\Delta }=1.45\times 10^{-3}\;m$ and ξ=5, comparison among dynamic analyses, elasto-static solutions and experimental data: (a) displacement histories $u_{2}(t)$ and $u_{12}(t)$ compared with the corresponding solutions of the elasto-static problem and experimental data of the displacement of the $2^{nd}$ and $12^{th}$ body segment at the end of the first crawl-substep, (b) velocity histories of the same selected DoFs, being τ, for both plots, the duration of the transient phase.

Homogeneous initial conditions were uploaded to the system of n−1 differential equations in the n−1 unknowns $u_{i}$ and the dynamics was—coherently with the quasi-static model— activated by means of the inelastic displacement $\tilde{\Delta }$ prescribed to the terminal proleg. By solving the system’s equations in the n−1 DoFs, from $u_{2}(t)$ to $u_{12}(t)$, the displacement and velocity histories could be obtained. By way of example, the time-dependent solutions $u_{2}$ and $u_{12}$, representing, the two ends of the system at the first sub-step before the detachment of the first Winkler-like spring, are illustrated in figure 13.

Literature evidence [28] shows that generally overdamped systems with a large damping ratio (i.e. $\xi_{1}>1$) can trace back caterpillars’ viscoelastic behaviour. Although we have not yet measured this viscosity parameter, we assume ξ=5 and we adopt a set of parameters physically plausible for the larva (some derived from the literature, others from considerations drawn from the experimental videos produced). From figure 13, one can observe that the transient phase of both displacement and velocity histories is very short, say τ, about approximately 0.3s, thus perfectly matching the experimental duration of a crawl-step (for instance, referring to the experimental findings depicted in figure 1b, the whole cycle lasts 1.2s for allowing the detachment of the 4 abdominal prolegs). Additionally, one can notice that all $u_{i}(t)$ recover almost immediately the static solution (see figure 13), thus proving that the dynamic effects can be actually negligible for capturing the essential aspects playing the key role in the locomotion’s system. The experimental data derived from the caterpillar locomotion (figure 1a) are reported for the second and twelfth body segments in figure 13a, showing that the results are approximately close to the theoretical elasto-static solutions. However, in addition to the adhesion characterization already performed, a complete calibration of stiffness and viscosity parameters is necessary to refine the model and achieve a perfect matching between the theoretical dynamic curve and the experimental data. Future studies will focus on this aspect to further improve the accuracy of the model.

## 4. Discussion and conclusions

With in mind applications for advanced bio-inspired soft robotics, we analysed the characteristic competition between adhesion and elasticity in caterpillar locomotion. By starting from detailed experimental observations, we formulated a biophysics-informed mathematical model that both faithfully replicates the movements of these larvae and explains how the two above-mentioned mechanical features effectively interplay to determine the crawl-cycle while maintaining it as stable and periodical.

To this purpose, by firstly assuming that inertial and dissipative effects could be ignored in describing the caterpillar locomotion, we employed a one-dimensional discrete mechanical model where the interaction between body segments’ elasticity and prolegs’ gripping on the substrate was ruled by the ratio of cuticle/muscle tissue’s stiffness and Winkler-like springs one, also accounting for the proleg-substrate adhesion. Furthermore, as highlighted by experimental evidences, locomotion was triggered by the activation of an inelastic muscle contraction that induces a stress distribution into the larva’s body, this in turn causing detachment of selected prolegs when the reaction forces they withstand achieve the adhesion threshold. By iterating step-by-step this mechanism up to all prolegs are detached, the crawl-cycle of the whole animal can be replicated, with the caterpillar that moves at the end of this process exactly of the initial prescribed inelastic (contraction-induced) displacement. As a result of the model, we also demonstrated that this inelastic displacement triggering locomotion can be inversely estimated as a function of both the stiffness ratio between body and prolegs and the adhesion threshold, by preserving the geometric compatibility of the system that *de facto* represents the necessary additional interpenetration constraint condition. In this manner, taking advantage of the fully parametric model, one can trace back the gait of several caterpillar species, each characterized by diverse morphomechanical features.

In this light, a meaningful direction for future work would be to use the results of our mechanical model, specifically the calibration of the inelastic contraction as a function of the stiffness ratio (see figure 9), to investigate whether real data from various caterpillar species are included in the admissible region. Biologists could measure these parameters over different species to assess whether they fall on or near this region. If confirmed, it would provide stronger evidence that the model captures essential biomechanical relationships relevant to the biology of the animals, potentially reflecting evolutionary optimization. On the contrary, if mechanical parameters deviate from the theoretical predictions, this would open intriguing questions about why the expected biomechanical relationships are not observed. Possible explanations could involve developmental constraints or trade-offs that prevent species from evolving to the predicted optimal states, offering an exciting avenue for further biological and ecological research.

At the end, by implementing a fully dynamic model in which both inertial and dissipative (viscous) effects were also incorporated, we proved that these dynamical features can be considered as negligible and the hypothesis of quasi-static caterpillar locomotion can be therefore confirmed, the characteristic times of the extinction of the transient phase being extremely short, as actually shown by the laboratory results. Notably, our experiments also revealed the ability of caterpillars to traverse wooden surfaces, highlighting the significance of the attachment/detachment process of prolegs in modulating the locomotive response. This unique and adaptive behaviour underlines the remarkable evolutionary optimization of caterpillar locomotion, stabilizing and controlling its efficient movement by exploiting the gripping on the supporting substrate.

We felt that this study, by unveiling the underlying mechanism ruling the caterpillar locomotion based on the competition between elasticity and adhesion, can pave the way for designing new soft robots with enhanced capabilities in moving on different material supports and geometrical surfaces.

Finally, it is worth noticing that while the current one-dimensional model is effective in capturing the main aspects of caterpillar locomotion, it may fall short in scenarios where lateral stability and Poisson effects in orthogonal directions are significant. Future developments could integrate these factors to enhance the model’s understanding of caterpillar biomechanics. Upcoming research will focus on expanding the model to include orthogonal degrees of freedom, large deformations and validating hypotheses and theoretical predictions through new key experimental data.

## Data Availability

The codes and data employed for the research have been provided at Dryad data repository: [57].
